# 764. Return to the Roundtable: A Clinical Case Discussion Curriculum for Infectious Diseases Fellows

**DOI:** 10.1093/ofid/ofad500.825

**Published:** 2023-11-27

**Authors:** Molly Hillenbrand, Reinaldo Perez, Christopher Shoff, Eileen K Maziarz, Matthew A Sparks

**Affiliations:** Duke University, Durham, North Carolina; Duke University, Durham, North Carolina; Duke University School of Medicine, DURHAM, North Carolina; Duke University Medical Center, Durham, NC; Duke University, Durham, North Carolina

## Abstract

**Background:**

The Accreditation Council for Graduate Medical Education (ACGME) and the Infectious Diseases Society of America (IDSA) require infectious diseases fellowship programs to include a core curriculum as part of their requirements for clinical training, though recommendations regarding content and structure are limited. Despite advances in online tools available for asynchronous learning in medical education, there remains a scarcity of published ready-to-teach content. We hypothesized that case-based clinical reasoning sessions covering core clinical topics would improve the attitudes and confidence of infectious diseases fellows.

**Methods:**

We conducted an informal needs assessment of current fellows, reviewed published recommendations and other fellowship curricula. Then, we developed a series of case-based clinical reasoning sessions for clinical and research fellows based on constructivist theory and emphasizing adult learning principles. Topics were rooted in published guidelines and evidence-based practice, prioritizing complex and high-yield clinical topics. Content was peer-reviewed by faculty and sessions were facilitated by senior fellows. Facilitation guides were created to allow for dissemination in the future. Faculty and pharmacists attended the sessions as expert discussants. Fellows completed anonymous surveys assessing attitudes and confidence after each session.

**Results:**

Three sessions covering Endemic Mycoses, Non-tuberculous Mycobacteria, and Toxoplasmosis were completed with 6-8 fellows in attendance at each. Surveyed fellows felt that the sessions were valuable and improved their confidence in clinical management of the topics presented (Figure 1). Participants also provided narrative feedback (Table 1).

Post-session Survey Results

**Figure 1.**
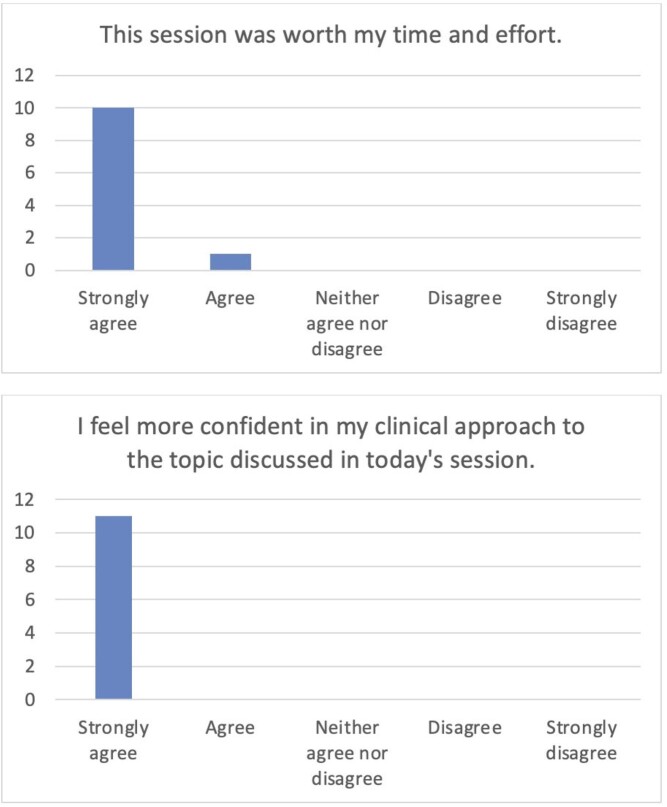
Post-session survey results from Likert-scale questions assessing attitudes and confidence, n=11 surveys completed by infectious diseases fellows.

Post-session Narrative Feedback

**Table 1.**
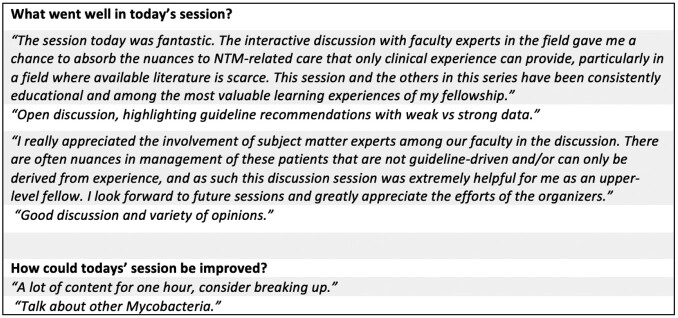
Narrative feedback from fellows who completed the post-session surveys.

**Conclusion:**

This curriculum represents a novel initiative by trainee physicians to create a series of peer-facilitated, discussion-based sessions for learners at the level of subspecialty fellowship. We demonstrated that these sessions improve the attitudes and confidence of fellows in a single-program pilot. Future directions will include studying the intervention in other fellowship programs and sharing this program broadly within the infectious diseases education community.

**Disclosures:**

**Eileen K. Maziarz, MD**, Karius, Inc: Advisor/Consultant

